# A large, curated, open-source stroke neuroimaging dataset to improve lesion segmentation algorithms

**DOI:** 10.1038/s41597-022-01401-7

**Published:** 2022-06-16

**Authors:** Sook-Lei Liew, Bethany P. Tavenner, Miranda R. Donnelly, Artemis Zavaliangos-Petropulu, Jessica N. Jeong, Giuseppe Barisano, Alexandre Hutton, Julia P. Simon, Julia M. Juliano, Anisha Suri, Zhizhuo Wang, Aisha Abdullah, Jun Kim, Tyler Ard, Nerisa Banaj, Michael R. Borich, Lara A. Boyd, Amy Brodtmann, Cathrin M. Buetefisch, Lei Cao, Jessica M. Cassidy, Valentina Ciullo, Adriana B. Conforto, Steven C. Cramer, Rosalia Dacosta-Aguayo, Ezequiel de la Rosa, Martin Domin, Adrienne N. Dula, Wuwei Feng, Alexandre R. Franco, Fatemeh Geranmayeh, Alexandre Gramfort, Chris M. Gregory, Colleen A. Hanlon, Brenton G. Hordacre, Steven A. Kautz, Mohamed Salah Khlif, Hosung Kim, Jan S. Kirschke, Jingchun Liu, Martin Lotze, Bradley J. MacIntosh, Maria Mataró, Feroze B. Mohamed, Jan E. Nordvik, Gilsoon Park, Amy Pienta, Fabrizio Piras, Shane M. Redman, Kate P. Revill, Mauricio Reyes, Andrew D. Robertson, Na Jin Seo, Surjo R. Soekadar, Gianfranco Spalletta, Alison Sweet, Maria Telenczuk, Gregory Thielman, Lars T. Westlye, Carolee J. Winstein, George F. Wittenberg, Kristin A. Wong, Chunshui Yu

**Affiliations:** 1 Chan Division of Occupational Science and Occupational Therapy, University of Southern California, Los Angeles, CA USA; 2 Mark and Mary Stevens Neuroimaging and Informatics Institute, Keck School of Medicine, University of Southern California, Los Angeles, CA USA; 3 Laboratory of Neuroimaging, Mark and Mary Stevens Neuroimaging and Informatics Institutes, Keck School of Medicine, University of Southern California, Los Angeles, CA USA; 4 Neuroscience Graduate Program, University of Southern California, Los Angeles, CA USA; 5 Electrical and Computer Engineering, Swanson School of Engineering, University of Pittsburgh, Pittsburgh, PA USA; 6 Laboratory of Neuropsychiatry, IRCCS Santa Lucia Foundation, Rome, Italy; 7 Department of Rehabilitation Medicine, Emory University School of Medicine, Atlanta, GA USA; 8 Department of Physical Therapy & Djavad Mowafaghian Centre for Brain Health, University of British Columbia, Vancouver, British Columbia Canada; 9 Florey Institute of Neuroscience and Mental Health, University of Melbourne, Melbourne, Victoria Australia; 10 Department of Neurology, Emory University, Atlanta, GA USA; 11 Center for the Developing Brain, Child Mind Institute, New York, NY USA; 12 Department of Allied Health Sciences, University of North Carolina at Chapel Hill, Chapel Hill, NC USA; 13 Hospital das Clínicas, São Paulo University, Sao Paulo, SP Brazil; 14 Hospital Israelita Albert Einstein, Sao Paulo, SP Brazil; 15 Department of Neurology, University of California Los Angeles and California Rehabilitation Institute, Los Angeles, CA USA; 16 Department of Psychiatry and Clinical Psychobiology, University of Barcelona, Barcelona, Spain; 17 icometrix, Leuven, Belgium; 18 Department of Computer Science, Technical University of Munich, Munich, Germany; 19 Functional Imaging Unit, Department of Diagnostic Radiology and Neuroradiology, University of Greifswald, Greifswald, Germany; 20 Departments of Neurology and Diagnostic Medicine, Dell Medical School at The University of Texas Austin, Austin, TX USA; 21 Department of Neurology, Duke University School of Medicine, Durham, NC USA; 22 Center for Biomedical Imaging and Neuromodulation, Nathan Kline Institute for Psychiatric Research, Orangeburg, NY USA; 23 Department of Psychiatry, NYU Grossman School of Medicine, New York, NY USA; 24 Department of Brain Sciences, Imperial College London, London, UK; 25 Center for Data Science, Université Paris-Saclay, Inria, Palaiseau France; 26 Department of Health Sciences & Research, Medical University of South Carolina, Charleston, SC USA; 27 Cancer Biology, Wake Forest School of Medicine, Winston Salem, NC USA; 28 Innovation, Implementation and Clinical Translation (IIMPACT) in Health, Allied Health and Human Performance, University of South Australia, Adelaide, South Australia Australia; 29 Ralph H Johnson VA Medical Center, Charleston, SC USA; 30 The Florey Institute of Neuroscience and Mental Health, Heidelberg, VIC Australia; 31 Neuroradiology, School of Medicine, Technical University Munich, München, Germany; 32 Department of Radiology, Tianjin Medical University General Hospital, Tianjin, China; 33 Department of Medical Biophysics, University of Toronto, Toronto, Ontario Canada; 34 Hurvitz Brain Sciences Program, Toronto, Ontario Canada; 35 Department of Clinical Psychology and Psychobiology, Institut de Neurociències, Universitat de Barcelona, Barcelona, Spain; 36 Institut de Recerca Sant Joan de Déu, 08950 Esplugues de Llobregat, Spain; 37 Jefferson Magnetic Resonance Imaging Center, Philadelphia, PA USA; 38 CatoSenteret Rehabilitation Center, SON, Norway; 39 Faculty of Health Sciences, Oslo Metropolitan University, Oslo, Norway; 40 Inter-university Consortium for Political and Social Research, University of Michigan, Ann Arbor, MI USA; 41 Facility for Education and Research in Neuroscience, Emory University, Atlanta, GA USA; 42 ARTORG Center for Biomedical Engineering Research, University of Bern, Bern, Switzerland; 43 Schlegel-University of Waterloo Research Institute for Aging, University of Waterloo, Waterloo, Ontario Canada; 44 Canadian Partnership for Stroke Recovery, Sunnybrook Research Institute, Toronto, Ontario Canada; 45 Department of Rehabilitation Sciences, Medical University of South Carolina, Charleston, SC USA; 46 Clinical Neurotechnology Laboratory, Dept. of Psychiatry and Neurosciences (CCM), Charité - Universitätsmedizin Berlin, Berlin, Germany; 47 Menninger Department of Psychiatry and Behavioral Sciences, Division of Neuropsychiatry, Baylor College of Medicine, Houston, TX USA; 48 Department of Physical Therapy and Neuroscience, Samson College of Health Sciences, St. Joseph’s University, Philadelphia, PA USA; 49 Department of Psychology, University of Oslo, Oslo, Norway; 50 NORMENT, Department of Mental Health and Addiction, Oslo University Hospital, Oslo, Norway; 51 Division of Biokinesiology and Physical Therapy of the Herman Ostrow School of Dentistry, University of Southern California, Los Angeles, CA USA; 52 Department of Neurology, Keck School of Medicine, University of Southern California, Los Angeles, CA USA; 53 Geriatrics Research, Education and Clinical Center, HERL, Department of Veterans Affairs, Pittsburgh, PA USA; 54 Departments of Neurology, PM&R, RNEL, CNBC, University of Pittsburgh, Pittsburgh, PA USA; 55 Department of Physical Medicine & Rehabilitation, Dell Medical School, University of Texas at Austin, Austin, TX USA; 56 Tianjin Key Laboratory of Functional Imaging, Tianjin Medical University General Hospital, Tianjin, China

**Keywords:** Stroke, Brain

## Abstract

Accurate lesion segmentation is critical in stroke rehabilitation research for the quantification of lesion burden and accurate image processing. Current automated lesion segmentation methods for T1-weighted (T1w) MRIs, commonly used in stroke research, lack accuracy and reliability. Manual segmentation remains the gold standard, but it is time-consuming, subjective, and requires neuroanatomical expertise. We previously released an open-source dataset of stroke T1w MRIs and manually-segmented lesion masks (ATLAS v1.2, N = 304) to encourage the development of better algorithms. However, many methods developed with ATLAS v1.2 report low accuracy, are not publicly accessible or are improperly validated, limiting their utility to the field. Here we present ATLAS v2.0 (N = 1271), a larger dataset of T1w MRIs and manually segmented lesion masks that includes training (n = 655), test (hidden masks, n = 300), and generalizability (hidden MRIs and masks, n = 316) datasets. Algorithm development using this larger sample should lead to more robust solutions; the hidden datasets allow for unbiased performance evaluation via segmentation challenges. We anticipate that ATLAS v2.0 will lead to improved algorithms, facilitating large-scale stroke research.

## Abstract (summary)

**Table:** 

Measurement(s)	stroke lesion
Technology Type(s)	manual segmentation in ITK-SNAP
Sample Characteristic - Organism	Homo sapiens
Sample Characteristic - Environment	brain

## Background & Summary

Large neuroimaging datasets are increasingly being used to identify novel brain-behavior relationships in stroke rehabilitation research^1,2^. Lesion location and lesion overlap with extant brain structures and networks of interest are consistently reported as key predictors of stroke outcomes^3–6^. However, in order to examine these measures in large datasets, accurate automated methods for detecting and delineating stroke lesions are needed. Two critical barriers limiting accurate automated segmentation in rehabilitation research are the variability in post-stroke neuroanatomy across patients and the limited amount of diverse data with which to train and test segmentation algorithms.

In acute stroke, large clinical neuroimaging datasets have led to improvements in segmentation algorithms for clinical MRI protocols (e.g., diffusion weighted imaging, FLAIR, or T2-weighted MRI)^7–9^. However, MRIs are not routinely collected as part of stroke rehabilitation clinical care, which usually commences at subacute or chronic stages. To obtain neuroimaging data at this stage, rehabilitation researchers often recruit people with stroke to participate in research studies, requiring significant time, funding effort and cost to generate even small datasets. In addition, high-resolution T1-weighted (T1w) MRIs are typically used at this stage to identify and delineate lesioned tissue, as T1w MRI provides excellent spatial resolution and is required for registering other research imaging data, such as functional MRI and diffusion MRI. Although other imaging modalities, such as T2-weighted MRI or FLAIR imaging, would be helpful for identifying additional white matter abnormalities, they are often not routinely collected. This is due to limited scanning time, which is allocated for MR sequences directly related to the researcher’s hypotheses. However, because lesions are often more challenging to identify at this later stage, and T1w single-channel imaging is incompatible with most multispectral tools developed for acute clinical imaging, there are few options for automated lesion segmentation. Of the existing automated lesion segmentation tools for single-channel, T1w MRI data, most are not highly accurate or reliable^10^ and require significant manual effort for quality control and correction^1^. Due to these challenges, manual lesion segmentation remains the gold standard in stroke rehabilitation research, but it is inefficient, subjective, and limits large-scale stroke rehabilitation research.

Machine learning, and in particular, deep learning algorithms, have been applied to address this problem, but they require large, diverse training datasets to create generalizable models that can perform well on new data. To this end, we previously released a public dataset of 304 stroke T1w MRIs and manually segmented lesion masks called the Anatomical Tracings of Lesions After Stroke (ATLAS) v1.2 dataset^11^. ATLAS is the largest dataset of its kind and intended to be a resource for the scientific community to develop more accurate lesion segmentation algorithms. It is also meant to be used as a standardized benchmark with which to compare the performance of different segmentation methods^10^. The data are derived from diverse, multi-site data from 11 research cohorts worldwide and harmonized by the ENIGMA Stroke Recovery working group^1^. ATLAS v1.2 has been accessed and cited widely since its release in 2018, with reports including the improved performance of stroke lesion segmentation algorithms using novel methods, particularly deep learning and convolutional neural networks (e.g.^12–28^).

The reach of the ATLAS v1.2 dataset has also extended beyond stroke lesion segmentation. It has also been used as a key example of a large, public neuroimaging dataset^29^, to provide published guidelines on how to perform lesion segmentation^30^, to evaluate the performance of different hippocampal segmentation methods in stroke^31^, to test other non-stroke automated methods, such as anomaly^32^ and asymmetry detection^33^, and as inspiration for future AI programs and large public datasets^34^, among other uses. It is a valuable educational resource and has been used as a teaching resource in courses on machine learning and computer vision as well as for student thesis projects. It has been cited by over 75 publications and downloaded over 1800 times from over 30 countries in the past several years since its release, demonstrating its significant global impact on the scientific and academic community.

However, while ATLAS v1.2 spurred the development of many new automated lesion segmentation methods (Table 1), there are still no publicly available automated methods that have reported performance reliable enough to be used for research. Although no published standards exist, in our own research we estimate that a minimum Dice coefficient, or measure of overlap between the true lesion and the predicted lesion mask^35^, of greater than 0.85 needs to be reached before a method can be declared sufficiently reliable to replace manual segmentation. In 2018, we used the ATLAS v1.2 dataset as a benchmark to evaluate publicly available automated lesion segmentation methods using T1w MRIs, but the best performing method (Lesion Identification with Neighborhood Data Analysis, or LINDA)^36^ only had an average Dice coefficient of 0.5 on ATLAS v1.2^10^. Similarly, all of the more recently published methods that were trained and tested on ATLAS v1.2 report an average Dice coefficient under 0.7 (see Table 1 for details). In addition, because ATLAS v1.2 is a completely public dataset, without a partitioned test dataset, it is possible for researchers to overfit their model, not perform proper validation, or incorrectly calculate the Dice coefficient. This can lead to artificially inflated performance metrics. ATLAS v1.2 did not contain separate test data, which is necessary to reliably evaluate algorithm performance and generalizability to new data. Finally, of the 17 different methods published using ATLAS v1.2, 12 papers did not report publicly available code, limiting their utility to the scientific community.

**Table 1:** Published Methods for Automated Lesion Segmentation Using ATLAS v1.2.

Article	Method	Reported Dice	Code Publicly Available	*n*	Validation Method	Input size 2D/3D (H, W, D)
					**Cross-validation**	
Basak *et al*., 2021	DFENet	0.546	no	229	5-fold cross-validation	2D 192, 192 or 3D 192, 192, 4
Hui *et al*., 2020	PSPF and U-Net	0.593	no	239	6-fold cross-validation	2D 176, 176
Lu *et al*., 2020	EDCL w/ 3D Unet	0.148 (0.584)**	no	239	5-fold cross-validation	3D 64, 64, 64
Qi *et al*., 2019	X-Net	0.487	yes	229	5-fold cross-validation	2D 192, 224
Zhang *et al*., 2020	MI-UNet	0.567	no	229	5-fold cross-validation	2D 233, 197 or 3D 49, 49, 49
					**One hold-out Train, Validation, Test**	
Chen *et al*., 2018	U-Net/GMM*	0.500/0.170	no	220	unclear/0, 0, 100 (%)	2D 128, 128 or 256, 256
Chen *et al*., 2020	VAE*/GMVAE*	0.110/0.120	no	220	0, 0, 100/0, 0, 100 (%)	2D 200, 200
Kervadec *et al*., 2020	Enet	0.474	yes	229	203, 26, 0	unclear
Liu *et al*., 2019	MSDF-Net	0.558	no	229	160, 69, 0	2D 224, 177
Paing *et al*., 2021	3D U-Net	0.668	no	239	60, 20, 20 (%)	3D 197, 233, 189
Qi *et al*., 2020	U-Net	0.518	no	229	120, 40, 69	2D 224, 192
Sahayam *et al*., 2020	MUDCap3	0.670	no	229	160, 69, 0	3D 256, 256, 256
Tomita *et al*., 2020	3D-ResU-Net	0.640	yes	239	76, 11, 13 (%)	3D 144, 172, 168
Wang *et al*., 2020	CPGAN	0.617	no	239	129, 40, 60	2D 256, 256
Xue *et al*., 2020	U-Net (9 paths)	0.540	yes	54	0, 0, 54	3D 192, 224, 192
Yang *et al*., 2019	CLCI-Net	0.581	yes	220	55, 18, 27 (%)	2D 224–233, 176–197
Zhou *et al*., 2019	D-Unet	0.535	no	229	80, 20, 0 (%)	2D 192, 192 or 3D 192, 192, 4

A summary of published automated lesion segmentation methods that were trained from ATLAS v1.2, with brief summaries of their method, validation method, and reported Dice coefficient. Blue rows indicate methods using cross-validation. Yellow rows indicate methods using one hold-out. *Indicates an out-of-distribution method that is trained only on non-lesioned images and detects outliers that possibly represent stroke lesions. **Indicates an incorrect equation for the Dice index computation; the correct Dice is 0.148 and the reported Dice is listed in parentheses.

To address the above-mentioned concerns, we created ATLAS v2.0, which expands upon and replaces ATLAS v1.2. ATLAS v2.0 contains 1271 T1w MRIs with manually segmented lesion masks from 44 different research cohorts across 11 countries worldwide (including ATLAS v1.2 data, which are denoted in the accompanying metadata).

ATLAS v2.0 improves on ATLAS v1.2 in several ways. First, it contains more than four times as much data as ATLAS v1.2 and from more diverse cohorts, providing a bigger dataset for training and testing. Second, ATLAS v2.0 provides a single lesion mask file that encompasses all detected lesions, instead of having separate files per lesion, which previous users reported as being cumbersome in ATLAS v1.2. Third, ATLAS v2.0 fixes minor errors and issues with registration and orientation noted in previous ATLAS releases. Finally, and most importantly, ATLAS v2.0 is split into three parts: (1) a training dataset, which is comprised of 655 publicly released T1w MRIs and lesion masks, (2) a test dataset, which is comprised of 300 publicly released T1w MRIs with hidden lesion masks, and (3) a generalizability dataset, which is comprised of 316 completely hidden T1w MRIs and lesion masks from separate cohorts. The hidden data is available only for testing algorithm performance in lesion segmentation challenges and competitions (see *Lesion Segmentation Challenges*). Notably, the training and test set contain similar distributions of data, such that an algorithm trained on the training set should perform well on the test set. However, the generalizability dataset of 316 cases (T1w MRI and lesion masks) are from completely new cohorts, and none of this data is publicly released in order to evaluate the generalizability of algorithm performance on completely unseen data. In these ways, we aim to reduce the risk of research groups overfitting their data and reporting inflated algorithm performance, with an overall goal of improving the state of the field. We also strongly encourage lesion segmentation challenges to require public sharing of submitted methods to facilitate greater scientific dissemination. In the current paper, we describe the ATLAS v2.0 dataset, along with several lesion segmentation challenge platforms that aim to utilize this dataset.

## Methods

### Data overview

Similar to our previous ATLAS v1.2 release, the ATLAS v2.0 dataset was aggregated from 44 research cohorts collected for various research purposes, with specific eligibility criteria, and therefore may not be representative of the general population of all patients with stroke. The general purposes of the research cohorts involved were to understand brain-behavior relationships between brain measures, functional outcomes (e.g., sensorimotor impairment, cognitive impairment, mood), and/or response to different therapies after stroke. In the case of intervention or observational studies with longitudinal data, only the first timepoint was included in ATLAS v2.0 (see also *Data Records)*. The data range from acute (within the first 24 hours after stroke) to chronic (more than 180 days after stroke); the time of MRI acquisition in relation to stroke onset is included in the metadata. The data are derived from studies that were approved by their local ethics committee and were conducted in accordance with the 1964 Declaration of Helsinki. Informed consent was obtained from all subjects. The ethics committee at the receiving site (the University of Southern California) approved the receipt and sharing of the de-identified data, which do not contain any personal identifiers.

**Fig. 1:**
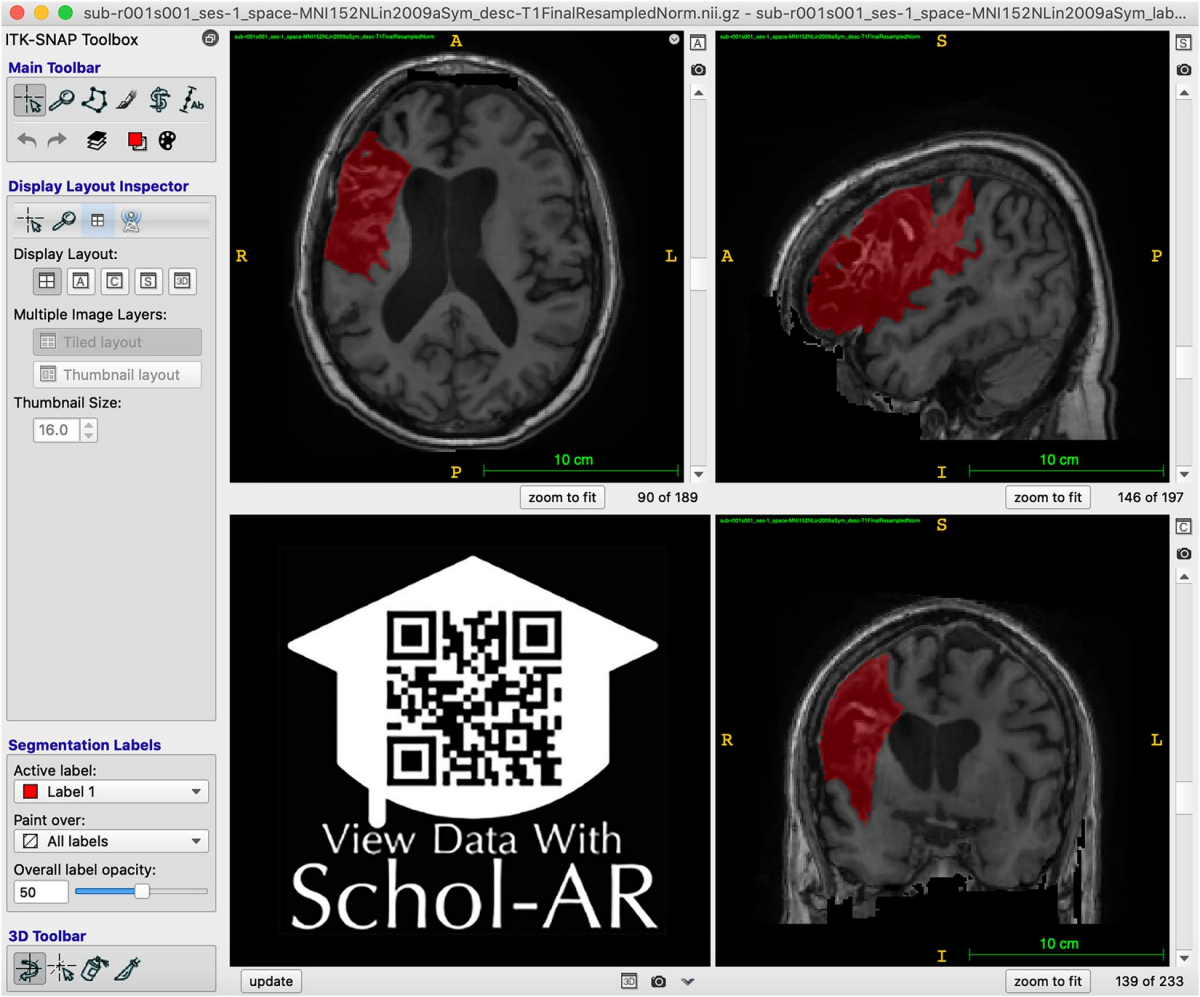
Example of Lesion Segmentation in ITK-SNAP. An example of the ITK-SNAP interface displaying a lesion segmentation mask (red) in radiological convention (the left hemisphere is shown on the right side of the screen). Axial (top left), sagittal (top right), and coronal (bottom right) planes are shown. A video of the example lesion mask in ITK-SNAP can be viewed through Schol-AR by scanning the QR code in the bottom left with a mobile device, or by opening this PDF with a non-mobile web browser at www.Schol-AR.io/reader.

For each subject file, we first performed quality control of the image. Images were excluded if large motion artifacts or other disruptions made it difficult to identify the lesion. Next, brain lesions were identified, and masks were manually drawn in native space. Our team identified and traced lesions using ITK-SNAP^37,38^ (version 3.8.0; Fig. 1; see lesion segmentation details below). After tracing, we reviewed and edited lesion masks as necessary using a standardized quality control protocol. In a subset of the data, lesion masks were received from the originating site and edited and checked for quality by our team. All team members received lesion-tracing training and followed a standard operating protocol for tracing lesions to ensure consistency across tracers^11^. All lesion masks were checked for quality by two separate trained team members. During the quality control process, we ensured that the boundaries of the lesion segmentation were accurate and that all identifiable lesions in the brain were traced.

**Fig. 2:**
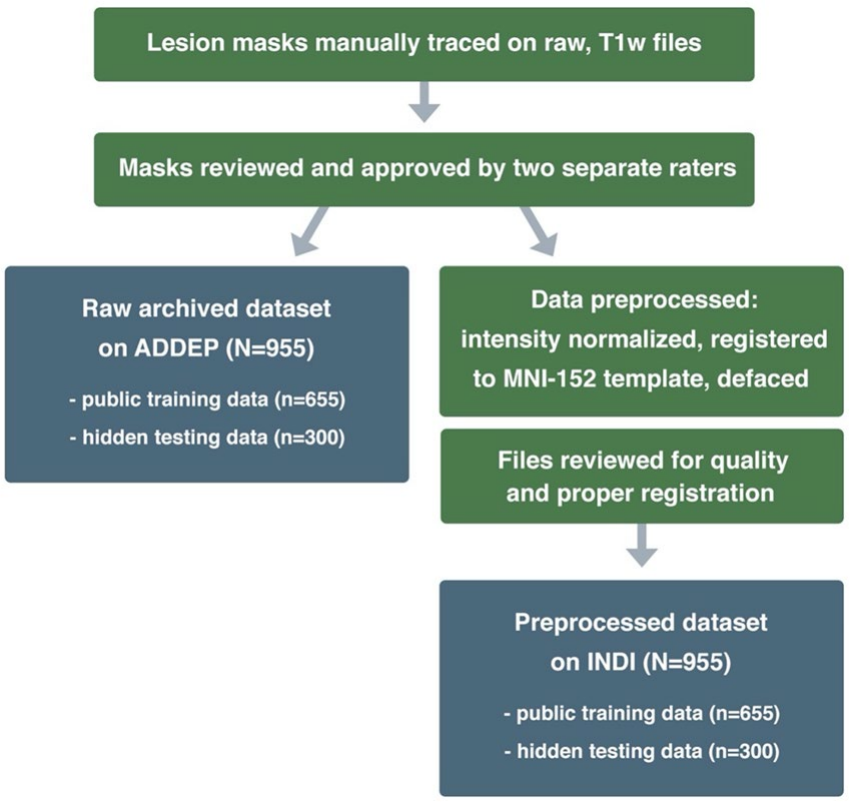
Lesion Tracing and Preprocessing Pipeline. A flowchart diagram demonstrating the process for creating the two archived datasets: a raw dataset in native space archived with the Archive of Data on Disability to Enable Policy and research (ADDEP) (left blue box) and a preprocessed dataset in MNI-152 space archived with the International Neuroimaging Data-Sharing Initiative (INDI) (right blue box).

ATLAS v2.0 includes all the same subjects as v1.2, with the removal of repeated subjects that had two timepoints (n = 9) so that in ATLAS v2.0, each subject is only represented once. All subject files have undergone a lesion tracing and preprocessing pipeline (Fig. 2) and are named and stored in accordance with the Brain Imaging Data Structure (BIDS) (http://bids.neuroimaging.io/)^39^. Metadata on scanner information, sample image headers for each cohort, and lesion information for each subject in the training dataset is included in the *Supplementary Materials*. The metadata also includes time of MRI acquisition in relation to stroke onset in days, where this data was available. However, subject demographic information, such as age, sex, or other clinical outcome measures, is not shared due to privacy concerns and data sharing policies at many of the contributing sites. We acknowledge that this information would greatly enhance the utility of this dataset; we aim to be able to include this information for at least a subset of data, where allowed, in future releases.

Of the 1271 samples, data from 955 samples were randomly split into public training and hidden test datasets across sites, so that the testing set includes a similar multi-site composition as the training set. As mentioned previously, lesion challenges will also have access to 316 samples from new cohorts in order to test the true generalizability of algorithms to completely unseen data. Finally, any previously released data used as part of ATLAS v1.2 was kept as part of the public training dataset to prevent contamination of the test dataset.

### Data characteristics

The T1w MRI data were collected on 1.5-Tesla and 3-Tesla MR scanners. All data are high-resolution (e.g., 1 mm^3^ or higher), with the exception of four cohorts who have at least one dimension with a resolution between 1–2 mm^3^ (R027, R047, R049, R050). Each cohort was collected on a single scanner using the same parameters except for 2 cohorts (R027, R049). In these cases, the metadata includes an example of each scanning parameter.

The entire dataset (N = 1271) is derived from 44 research cohorts in total. The training (n = 655) and test (n = 300) datasets are derived from the same 33 research cohorts; samples from each cohort are randomly assigned to either training or test datasets so that they will have similar compositions. Thus, algorithms trained on the training dataset should perform well on the test dataset. The generalizability dataset (n = 316) is derived from 11 new cohorts to test the performance of trained algorithms on completely unseen data.

During the review process for each lesion mask, metadata on number of lesions and lesion location (left vs. right hemisphere, cortical vs. subcortical) was manually recorded by a trained team member. This detailed information for each subject can be helpful for sorting the data into subgroups with different lesion characteristics. In the training dataset (n = 655), 61.9% of subjects had only a single lesion, and 38.1% had multiple lesions. Of the total subjects with multiple lesions, 7.2% had multiple lesions contained in either the left or right hemispheres only (noted as “Unilateral”), 18.5% had multiple lesions in both hemispheres (noted as “Bilateral”) and 12.4% had multiple lesions with at least one lesion in either the cerebellum or brainstem (noted as “Other”) (Table 2). Lesions were counted as separate and additional if they were non-contiguous with any other lesion. Lesions were nearly equally distributed between left and right hemispheres, with 57.1% of subjects exhibiting at least one left hemisphere lesion, 58.8% exhibiting one right hemisphere lesion, and 22.9% with one lesion in either the cerebellum or brainstem (noted as “Other”). Lesions were also documented as either subcortical, cortical and white matter, or other. Consistent with the criteria used for ATLAS v1.2, lesions defined as subcortical were contained completely within the white matter and subcortical structures. Lesions defined as cortical and/or white matter indicate that the lesions extend into the cortex; these lesions often also include white matter and/or subcortical structures. Finally, the “Other” category encompasses lesions falling in the brainstem or cerebellum. Among all lesions in the training dataset, 25.5% were cortical and/or in the white matter, 59.7% subcortical, and 14.8% other (Table 3). Corresponding metadata includes this information on lesion number and location for each subject in the training dataset.

**Table 2:** Lesion number and hemisphere location per subject.

	Subjects with One Lesion			Subjects with Multiple Lesions		
	Left	Right	Other	Unilateral	Bilateral	Other
**Training data (n** = **655)**	173 (26.4%)	187 (28.5%)	46 (7.0%)	47 (7.2%)	121 (18.5%)	81 (12.4%)
**Testing data (n** = **300)**	88 (29.3%)	95 (31.7%)	23 (7.7%)	16 (5.3%)	43 (14.3%)	35 (11.7%)

The number of subjects with one lesion or multiple lesions, subdivided into specific areas (left, right, other) is shown for the training and testing datasets (955 subjects in total).

**Table 3:** Lesion location (subcortical vs. cortical).

	Cortical and White Matter Lesions		Subcortical Lesions		Other	Total Lesions
	Left	Right	Left	Right		
**Training data (n** = **655)**	132 (12.0%)	149 (13.5%)	333 (30.2%)	324 (29.4%)	163 (14.8%)	1101
**Testing data (n** = **300)**	65 (14.3%)	80 (17.7%)	119 (26.3%)	115 (25.4%)	74 (16.3%)	453

The number of lesions identified in specific regions (cortical, subcortical, or other), separated by hemisphere, is shown for the training and testing datasets (955 subjects in total). Note that subjects could have multiple lesions, thus resulting in a total number of lesions that is greater than the total number of subjects.

We also provide time of MRI acquisition relative to stroke onset in the metadata in days in a column labeled “days post stroke.” In some cases, the exact number of days between stroke onset and MRI acquisition was not recorded or provided to us. For these participants, a general timeline was included (i.e., MRI was acquired greater than 180 days post-stroke); this was recorded in a column labeled “chronicity” where 180 indicates they are equal to or greater than 180 days post-stroke. Of note, several records did not have this accompanying information, so they have been marked as “NA”. However, we have provided as much data as possible to help inform the evaluation of algorithm performance based on time after stroke.

Metadata information is not provided for individual subjects within the test dataset (n = 300) to avoid biasing algorithms. However, it is presented at a group level. The test dataset is derived from 24 cohorts. Overall, 68.7% of subjects had only a single lesion and 31.3% had multiple lesions. Of the subjects with multiple lesions, 5.3% were marked “Unilateral”, 14.3% were marked “Bilateral”, and 11.7% were marked “Other” (Table 2). Lesions were nearly equally distributed between left and right hemispheres, with 51.7% of subjects exhibiting at least one left hemisphere lesion, 56.3% with at least one right hemisphere lesion, and 22.3% with at least one lesion in either the cerebellum or brainstem (noted as “Other”). Lesions were also documented as either subcortical, cortical, or other (existing in the cerebellum or brainstem). Among all lesions in the testing dataset, 32.0% were cortical and/or in the white matter, 51.7% subcortical, 16.3% other (Table 3). Data characteristics between the training and test datasets were similar.

Finally, metadata is not provided at all for the generalizability dataset (n = 316) to maintain its purpose of evaluating algorithm performance on unseen and unknown data. However, we note that it is comprised of multi-site data collected for research purposes, similar to the training and test datasets.

### Training for individuals performing lesion tracing

The research team responsible for the lesion segmentation and quality control followed the same training procedure to the training for the team that created ATLAS v1.2^11^, with the exception of using ITK-SNAP instead of MRIcron, due to its semi-automated lesion interpolation tool. Training for the lesion identification and tracing process involved study of in-depth neuroanatomy, standardized protocols, instructional videos, and consultations with a neuroradiologist. This protocol includes tracing the same initial set of lesions twice per person, with extensive feedback provided from multiple team members. Our standard operating procedures are freely available online (https://github.com/npnl/ATLAS/). The training manual for ITK-SNAP^37^ is freely available (http://www.itksnap.org/docs/fullmanual.php) and was also used as part of the lesion tracing process.

### Identifying and tracing lesions

For lesion identification, each T1w MRI was opened with ITK-SNAP (Fig. 1) and examined carefully. Tracers also received training in the identification of white matter hyperintensities of presumed vascular origin^40^ and perivascular spaces, which were excluded from the lesion masks as much as possible. Lesions were traced using either a mouse or stylus (i.e., Wacom Intuos Draw). All identified lesions for each subject were contained in a single image file. For lesions spanning a large number of slices (i.e., >50 slices), the “interpolation” tool was used. Upon completion, raw lesion mask files were saved and named according to a BIDS-compliant naming scheme (see also *Data Records)*.

All files were subsequently reviewed for quality control by two additional trained team members. If changes were necessary, edits were conducted by the original tracer. Upon approval, each subject’s raw mask and T1w image were added to the raw/native space dataset, then preprocessed and added to the preprocessed dataset. We recognize that manual tracing is a highly subjective process, even across similarly trained individuals, and we aimed to reduce any amount of tracing differences between tracers through multiple quality control steps. In the current release, we prioritized generating the largest possible dataset for public archiving. However, in a future release, we hope to also release a subset of the data with multiple lesion segmentation masks generated by different tracers. These multiple human ratings for each stroke brain could help establish a baseline for inter-rater variability, given the subjectivity of the task as noted above.

### Preprocessing normalization, registration and defacing

**Fig. 3:**
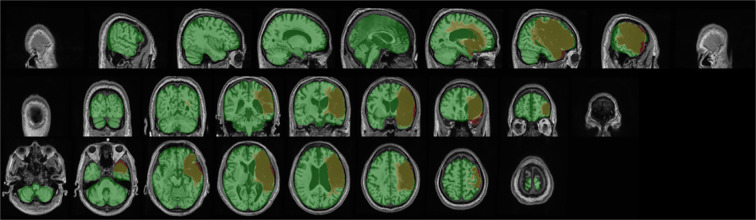
Example of Visual Quality Control. Example of an image used to ensure proper registration of each subject’s brain (gray) and lesion segmentation mask (reddish brown) to the MNI template (green).

In addition to releasing a dataset in native space with no preprocessing (raw; see *Data Records* below), we also released a preprocessed dataset that is archived with the International Neuroimaging Data-Sharing Initiative (INDI; Fig. 2). Each step in the preprocessing pipeline is identical to ATLAS v1.2, ensuring consistency across ATLAS versions. The pipeline includes intensity normalization and registration to a standardized template. In order to fully de-identify images, we also removed any potentially identifying non-brain data, such as facial images (termed defacing), a common procedure required to fully anonymize an MR brain image. First, we corrected for intensity non-uniformity and performed an intensity standardization step, which was completed with scripts included in the MINC-toolkit (https://github.com/BIC-MNI/minc-toolkit). After this correction, we used MINC tools to linearly register both T1w and lesion segmentation images to an MNI-152 template, which is included in the archive. Finally, we defaced the T1w images using the “mri_deface” tool from FreeSurfer (v1.22) (https://surfer.nmr.mgh.harvard.edu/fswiki/mri_deface). Per BIDS derivatives specifications, the T1w image and corresponding lesion mask are archived with file names of “sub-r***s***_ses-1_space-MNI152NLin2009aSym_T1w.nii.gz” and “sub-r***s***_ses-1_space-MNI152NLin2009aSym_label-L_desc-T1lesion_mask.nii.gz”, respectively (see also *Data Records* below for more details). Images that were previously excluded from ATLAS v1.2 due to errors in registration^11^ have now been included after manually correcting and inspecting them. After completion of the preprocessing pipeline, all subject files were visually inspected for quality to ensure correct lesion mask alignment and proper registration to the template (Fig. 3).

### Probabilistic spatial mapping of lesion location

**Fig. 4:**
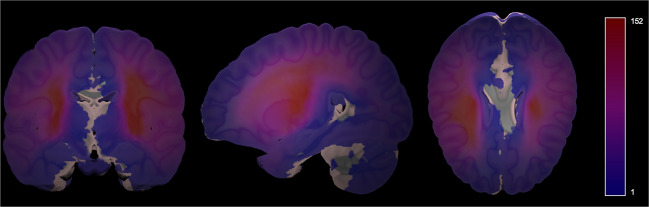
Probabilistic Lesion Overlap Map on the MNI_icbm152 Template. Visualization of the lesion overlap across all subjects (N = 955) overlaid on the MNI template, with hotter colors representing more subjects with lesions at that voxel. An interactive volumetric 3D display of this data may be viewed through Schol-AR by scanning the QR code from Fig. 1 with a mobile device, or by opening this PDF with a non-mobile web browser at www.Schol-AR.io/reader.

To visualize the average distribution of lesions contained in ATLAS v2.0 across the whole brain, we created a probabilistic map of lesions in the public stroke brains from the ATLAS v2.0 training and testing datasets with the MNI template (Fig. 4). This was completed with the *mincaverage* tool found in the MINC-toolkit (https://github.com/BIC-MNI/minc-toolkit). As noted previously, this may not be representative of all strokes and is only meant to visually demonstrate the voxels identified most commonly as lesioned in our dataset. This map has also been provided in NifTI format and uploaded to NeuroVault.org, where it can be freely accessed (https://neurovault.org/images/706022/).

## Data Records

Data are publicly available in preprocessed format (standardized to MNI-152 space) on INDI^41^ (fcon_1000.projects.nitrc.org/indi/retro/atlas.html), a free platform for neuroimaging data sharing. Raw data in native space are available on the Archive of Data on Disability to Enable Policy and research^42^ (ADDEP, 10.3886/ICPSR36684.v4), which has a more stringent restricted data use agreement to maintain privacy of the raw data. The metadata denotes whether each subject in the training dataset was previously part of the ATLAS v1.2 release. For the test dataset (n = 300), only the T1w scans, without lesion masks, are released on each platform so that users can test their algorithms on this data and submit their output to lesion segmentation challenges for evaluation. The generalizability dataset (n = 316) is only available for lesion segmentation challenges (see *Lesion Segmentation Challenges* below). None of the subjects from the previous ATLAS v1.2 release are included in either the test or generalizability datasets.

Data are maintained in BIDS format^39^. There are 33 cohorts in the training and testing datasets, and within each cohort folder are individual subject folders. We used the following naming convention: sub-r***s*** where r*** represents the research cohort number and s*** represents the subject number. All data are cross-sectional and from a single timepoint, so they all are denoted with “ses-1”. Native space images are labeled as “space-orig” while images normalized to the MNI-152 template are labeled as “space-MNI152Nlin2009aSym”. Finally, the description denotes that the lesion mask was traced from the T1w MRI (versus a different imaging modality, such as FLAIR).

Following BIDS conventions, a lesion mask in native space would be named as such: “sub-r***s***_ses-1_space-orig_label-L_desc-T1lesion_mask.nii.gz” and the corresponding T1w MRI would be named as “sub-r***s***_ses-1_space-orig_T1w.nii.gz.” As noted previously, the T1w MRI and lesion mask in MNI space are noted as: “sub-r***s***_ses-1_space-MNI152NLin2009aSym_T1w.nii.gz” and “sub-r***s***_ses-1_space-MNI152NLin2009aSym_label-L_desc-T1lesion_mask.nii.gz”, respectively.

## Technical Validation

The ATLAS v2.0 dataset was developed using similar protocols and methods as the ATLAS v1.2 dataset, which has been successfully utilized to develop numerous lesion segmentation methods for the last several years^12–28^. For ATLAS v2.0, detailed manual quality control for image quality occurred during the initial lesion segmentation, and all segmentations were examined for quality by two additional researchers. Following preprocessing, lesions were again checked for proper registration to template space. The ATLAS v2.0 dataset has been validated and incorporated into several new lesion segmentation challenges (see Lesion Segmentation Challenges below).

## Usage Notes

Data can be accessed under a standard Data Use Agreement, which requires users to agree to use the data only for research or statistical purposes, and not for the investigation of specific research subjects. Users of the ATLAS v2.0 dataset should properly acknowledge the data contributions of the authors and laboratories by citing this article and the specific data repository from which they accessed the data.

As previously noted, manual lesion segmentation can be subjective, and despite our extensive quality control process, errors can still occur. Any issues or feedback can be submitted on the ATLAS Github page under ‘issues’, which will be addressed by our research team (https://github.com/npnl/ATLAS/). Any changes to the data or updates with new data will be released under new ATLAS versions (e.g., v2.1, v2.2), and changes will be posted on Github.

Finally, to accompany ATLAS v2.0, we also have released updated open-source software for analyzing lesions (Pipeline for Analyzing Lesions After Stroke (PALS))^28^. This software allows users to calculate lesion volume, evaluate lesion overlap with brain regions of interest, and create lesion overlap images (similar to that shown in Fig. 4; see *Code Availability*).

### Lesion segmentation challenges

A key purpose of the ATLAS v2.0 dataset is to provide hidden test data to fairly evaluate the performance of lesion segmentation algorithms. To this end, the ATLAS v2.0 lesion mask test data (n = 300) and generalizability dataset (n = 316 T1w MRIs and lesion masks) are only available for lesion segmentation challenges upon request to the corresponding author. The ideal challenge will provide fast, web-based evaluation, share results on a public leaderboard, and will require public sharing of submitted algorithms with clear usage instructions to advance scientific knowledge within the community and continually improve on the best available algorithms.

Following our ATLAS v1.2 release, we found that a large percentage of users of the ATLAS dataset are students from around the world who used this data to learn how to apply machine learning, deep learning, and/or computer vision methods to this challenging problem. ATLAS v1.2 was used widely for student theses and class projects, as well as for training individuals in algorithm development, and we anticipate that ATLAS v2.0 will be used extensively for these purposes as well. Given the educational interest in ATLAS, a challenge using the ATLAS v2.0 data has been established through a partnership with the Paris-Saclay Center for Data Science using their Rapid Analytics and Model Prototyping (RAMP) project management tool (https://paris-saclay-cds.github.io/ramp-docs/)^43^. RAMP challenges are open and collaborative web challenges that provide informative starter kits in Python to reduce the barrier of entry for participants^43^. The starter kits provide background information on the problem as well as basic solution code. The RAMP approach democratizes science by allowing novice data scientists and learners to approach new technical problems by providing the foundational knowledge necessary to get started in the field and giving everyone the same starting point. RAMP challenges consist of a competitive phase, during which participants work individually to solve the problem, and a collaborative phase, during which participants can see each other’s solutions and work together to create the best final solution. Following the competitive phase, participants submit their solutions and code to the RAMP website, where they can see the results of everyone’s submissions. Because code is openly shared in the collaborative phase, participants can learn from one another’s solutions and work together to develop the best combined solution. This collaborative method has been used to successfully address over 20 different scientific challenges and is an excellent educational tool^43^. More information about the RAMP automated lesion segmentation challenge using ATLAS v2.0 data can be found here: https://ramp.studio/problems/stroke_lesions. This RAMP challenge may also be made available for use by course instructors and can provide a project platform for collaborative learning at events such as Brainhacks, which bring together scientists around the world to work together on challenging brain imaging problems^44^.

ATLAS v2.0 is also part of the Ischemic Stroke Lesion Segmentation (ISLES) Challenge at the International Conference on Medical Image Computing and Computer Assisted Intervention (MICCAI) in 2022 (http://www.isles-challenge.org/). The ISLES challenge is one of the best-known stroke lesion segmentation challenges and has attracted hundreds of researchers to the competition over the years to showcase the performance of novel methods. The ISLES challenge series started in 2015 and has taken place at MICCAI for multiple years, incorporating new datasets and clinical and technical challenges each year^9^. ISLES datasets often serve as benchmarks for the field, and teams are invited to submit their algorithms for publication following the challenge^9,45,46^. Adding ATLAS v2.0 to the ISLES challenge introduces stroke data across acute to chronic timepoints into the challenge for the first time and presents a unique single-channel (versus multispectral) imaging challenge. The ISLES 2022 challenge utilizes both ATLAS v2.0 test and generalizability datasets for algorithm evaluation via the Grand Challenge platform (https://atlas.grand-challenge.org/). Importantly, this platform will be used to publicly and automatically evaluate algorithm performance both during ISLES 2022 and after, for ongoing public evaluation. http://www.isles-challenge.org/We also include an accompanying sample solution on Github to assist users in getting started (see *Code Availability*).

ENIGMA Stroke Recovery receives new stroke MRI data on an ongoing basis, and we continually generate lesion segmentations that can be used as additional test data. New cohort data may be added to our generalizability dataset and used only in lesion segmentation challenges (e.g., expanding on our current n = 316 completely hidden test dataset), and we anticipate sharing additional data in future ATLAS releases. In future challenges, data may also be sorted into small, medium and large lesions, as we previously showed that automated methods performed the worst on small, followed by medium, lesions, and perform the best on large lesions^10^. This is likely due to the ease of detection of large lesions boundaries, whereas small lesions can often be missed completely or mistaken for other brain pathology^10^. Future challenges may focus on accurate identification of small lesions only, or on improving the accuracy of medium and large lesion segmentation boundaries.

In conclusion, ATLAS v2.0 builds on our previous ATLAS v1.2 release and provides a total archive of 1271 images, including 955 public images, separated into 655 public training cases and 300 test cases, and 316 completely unseen images from new cohorts available only for lesion segmentation challenges. Our primary goal in releasing ATLAS v2.0 is to enable the development of more accurate, robust and generalizable lesion segmentation algorithms using single-channel T1-weighted MR images. We anticipate that the larger sample size, hidden test dataset, generalizability dataset, and collaboration with lesion segmentation challenge platforms will lead to the development of improved lesion segmentation algorithms. The ultimate goal of this work is to increase the reproducibility of stroke MRI studies and facilitate large-scale stroke neuroimaging analyses to inform stroke rehabilitation research.

## Data Availability

The ATLAS v2 lesion segmentations were generated using ITK-SNAP version 3.8.0. Our protocols for lesion segmentation can be found on our Github (https://github.com/npnl/atlas). Code used to preprocess the dataset were adapted from the MINC-toolkit (https://github.com/BIC-MNI/minc-toolkit). T1w images were defaced using the “mri_deface” tool from FreeSurfer (v1.22) (https://surfer.nmr.mgh.harvard.edu/fswiki/mri_deface). PALS, our open-source software to perform lesion analyses, can be accessed at https://github.com/npnl/PALS. Finally, as part of the MICCAI ISLES 2022 challenge, we provide sample code on our Github (https://github.com/npnl/isles_2022/) to assist users in getting started with the lesion segmentation challenge (e.g., code to obtain the data, load it, and save predictions in the format expected by our automatic evaluator).

## References

[1] Liew, S.-L. *et al*. The ENIGMA Stroke Recovery Working Group: Big data neuroimaging to study brain-behavior relationships after stroke. *Human brain mapping* **43**, 129–148, 10.1002/hbm.25015 (2022). 32310331 10.1002/hbm.25015 PMC8675421

[2] Liew, S.-L. *et al*. Smaller spared subcortical nuclei are associated with worse post-stroke sensorimotor outcomes in 28 cohorts worldwide. *Brain Communications*, 10.1093/braincomms/fcab254 (2021). 10.1093/braincomms/fcab254 PMC8598999 34805997

[3] Boyd, L. A. *et al*. Biomarkers of stroke recovery: Consensus-based core recommendations from the Stroke Recovery and Rehabilitation Roundtable. *Neurorehabilitation and neural repair* **31**, 864–876 (2017). 29233071 10.1177/1545968317732680

[4] Feng, W. *et al*. Corticospinal tract lesion load: An imaging biomarker for stroke motor outcomes. *Ann Neurol* **78**, 860–870, 10.1002/ana.24510 (2015). 26289123 10.1002/ana.24510 PMC4715758

[5] Kim, B. & Winstein, C. Can neurological biomarkers of brain impairment be used to predict poststroke motor recovery? A systematic review. *Neurorehabilitation and neural repair* **31**, 3–24 (2017). 27503908 10.1177/1545968316662708

[6] Cassidy, J. M., Tran, G., Quinlan, E. B. & Cramer, S. C. Neuroimaging identifies patients most likely to respond to a restorative stroke therapy. *Stroke* **49**, 433–438 (2018). 29321336 10.1161/STROKEAHA.117.018844 PMC5780222

[7] Chen, L., Bentley, P. & Rueckert, D. Fully automatic acute ischemic lesion segmentation in DWI using convolutional neural networks. *NeuroImage: Clinical* **15**, 633–643 (2017). 28664034 10.1016/j.nicl.2017.06.016 PMC5480013

[8] Wu, O. *et al*. Big data approaches to phenotyping acute ischemic stroke using automated lesion segmentation of multi-center magnetic resonance imaging data. *Stroke* **50**, 1734–1741 (2019). 31177973 10.1161/STROKEAHA.119.025373 PMC6728139

[9] Maier, O. *et al*. ISLES 2015-A public evaluation benchmark for ischemic stroke lesion segmentation from multispectral MRI. *Medical image analysis* **35**, 250–269 (2017). 27475911 10.1016/j.media.2016.07.009 PMC5099118

[10] Ito, K. L., Kim, H. & Liew, S. L. A comparison of automated lesion segmentation approaches for chronic stroke T1‐weighted MRI data. *Human brain mapping* **40**, 4669–4685 (2019). 31350795 10.1002/hbm.24729 PMC6851560

[11] Liew, S.-L. *et al*. A large, open source dataset of stroke anatomical brain images and manual lesion segmentations. *Scientific data* **5**, 180011 (2018). 29461514 10.1038/sdata.2018.11 PMC5819480

[12] Paing, M. P., Tungjitkusolmun, S., Bui, T. H., Visitsattapongse, S. & Pintavirooj, C. Automated Segmentation of Infarct Lesions in T1-Weighted MRI Scans Using Variational Mode Decomposition and Deep Learning. *Sensors* **21**, 1952 (2021). 33802223 10.3390/s21061952 PMC7999810

[13] Xue, Y. *et al*. A multi-path 2.5 dimensional convolutional neural network system for segmenting stroke lesions in brain MRI images. *NeuroImage: Clinical* **25**, 102118 (2020). 31865021 10.1016/j.nicl.2019.102118 PMC6931186

[14] Qi, K. *et al*. In *International conference on medical image computing and computer-assisted intervention*. 247–255 (Springer).

[15] Zhou, Y., Huang, W., Dong, P., Xia, Y. & Wang, S. D-UNet: a dimension-fusion U shape network for chronic stroke lesion segmentation. *IEEE/ACM transactions on computational biology and bioinformatics* (2019). 10.1109/TCBB.2019.2939522 31502985

[16] Yang, H. *et al*. In *International Conference on Medical Image Computing and Computer-Assisted Intervention*. 266–274 (Springer).

[17] Chen, X., You, S., Tezcan, K. C. & Konukoglu, E. Unsupervised lesion detection via image restoration with a normative prior. *Medical image analysis* **64**, 101713 (2020). 32492582 10.1016/j.media.2020.101713

[18] Tomita, N., Jiang, S., Maeder, M. E. & Hassanpour, S. Automatic post-stroke lesion segmentation on MR images using 3D residual convolutional neural network. *NeuroImage: Clinical* **27**, 102276 (2020). 32512401 10.1016/j.nicl.2020.102276 PMC7281812

[19] Basak, H., Hussain, R. & Rana, A. DFENet: A Novel Dimension Fusion Edge Guided Network for Brain MRI Segmentation. *arXiv preprint arXiv:2105.07962* (2021).

[20] Chen, X., Pawlowski, N., Rajchl, M., Glocker, B. & Konukoglu, E. Deep generative models in the real-world: An open challenge from medical imaging. *arXiv preprint arXiv:1806.05452* (2018).

[21] Hui, H., Zhang, X., Li, F., Mei, X. & Guo, Y. A partitioning-stacking prediction fusion network based on an improved attention U-Net for stroke lesion segmentation. *IEEE Access* **8**, 47419–47432 (2020).

[22] Kervadec, H., Dolz, J., Wang, S., Granger, E. & Ayed, I. B. in *Medical Imaging with Deep Learning*. 365–381 (PMLR).

[23] Liu, X. *et al*. MSDF-Net: Multi-scale deep fusion network for stroke lesion segmentation. *IEEE Access* **7**, 178486–178495 (2019).

[24] Lu, Y., Zhou, J. H. & Guan, C. In 2020 *42nd Annual International Conference of the IEEE Engineering in Medicine & Biology Society (EMBC)*. 1059–1062 (IEEE). 10.1109/EMBC44109.2020.9176663 33018168

[25] Qi, K. *et al*. Multi-task MR Imaging with Iterative Teacher Forcing and Re-weighted Deep Learning. *arXiv preprint arXiv:2011.13614* (2020).

[26] Sahayam, S., Abirami, A. & Jayaraman, U. In *2020 IEEE 4th Conference on Information & Communication Technology (CICT)*. 1–6 (IEEE).

[27] Wang, S., Chen, Z., Yu, W. & Lei, B. Brain Stroke Lesion Segmentation Using Consistent Perception Generative Adversarial Network. *arXiv preprint arXiv:2008.13109* (2020).

[28] Zhang, Y. *et al*. MI-UNet: multi-inputs UNet incorporating brain parcellation for stroke lesion segmentation from T1-weighted magnetic resonance images. *IEEE Journal of Biomedical and Health Informatics* **25**, 526–535 (2020). 10.1109/JBHI.2020.2996783 32750908

[29] Deng, L. *et al*. The SUSTech-SYSU dataset for automatically segmenting and classifying corneal ulcers. *Scientific data* **7**, 1–7 (2020). 31959768 10.1038/s41597-020-0360-7 PMC6971241

[30] Boyne, P. *et al*. Functional magnetic resonance brain imaging of imagined walking to study locomotor function after stroke. *Clinical Neurophysiology* **132**, 167–177 (2021). 33291023 10.1016/j.clinph.2020.11.009 PMC7856193

[31] Zavaliangos-Petropulu, A. *et al*. Testing a convolutional neural network-based hippocampal segmentation method in a stroke population. *BioRxiv* (2020). 10.1002/hbm.25210 PMC8675423 33067842

[32] Martins, S. B., Falcao, A. X. & Telea, A. C. In *BIOIMAGING*. 74–81.

[33] Martins, S. B., Ruppert, G., Reis, F., Yasuda, C. L. & Falcão, A. X. In *2019 IEEE 16th Inte*rnatio*nal Symposium on Biomedical Imaging (ISBI 2019)*. 882–885 (IEEE).

[34] Yeo, M. *et al*. Artificial intelligence in clinical decision support and outcome prediction–applications in stroke. *Journal of medical imaging and radiation oncology* (2021). 10.1111/1754-9485.13193 34050596

[35] Crum, W. R., Camara, O. & Hill, D. L. Generalized overlap measures for evaluation and validation in medical image analysis. *IEEE transactions on medical imaging* **25**, 1451–1461 (2006). 17117774 10.1109/TMI.2006.880587

[36] Pustina, D. *et al*. Automated segmentation of chronic stroke lesions using LINDA: Lesion identification with neighborhood data analysis. *Human brain mapping* **37**, 1405–1421, 10.1002/hbm.23110 (2016). 26756101 10.1002/hbm.23110 PMC4783237

[37] Yushkevich, P. A. & Gerig, G. ITK-SNAP: an intractive medical image segmentation tool to meet the need for expert-guided segmentation of complex medical images. *IEEE pulse* **8**, 54–57 (2017). 28715317 10.1109/MPUL.2017.2701493

[38] Yushkevich, P. A. *et al*. User-guided 3D active contour segmentation of anatomical structures: significantly improved efficiency and reliability. *NeuroImage* **31**, 1116–1128 (2006). 16545965 10.1016/j.neuroimage.2006.01.015

[39] Gorgolewski, K. J. *et al*. The brain imaging data structure, a format for organizing and describing outputs of neuroimaging experiments. *Scientific Data* **3**, 160044 (2016). 27326542 10.1038/sdata.2016.44 PMC4978148

[40] Wardlaw, J. M. *et al*. Neuroimaging standards for research into small vessel disease and its contribution to ageing and neurodegeneration. *The Lancet Neurology* **12**, 822–838 (2013). 23867200 10.1016/S1474-4422(13)70124-8 PMC3714437

[41] Liew, S.-L. *et al*. Anatomical Tracings of Lesions After Stroke (ATLAS) R2.0. *International Neuroimaging Data-Sharing Initiative* fcon_1000.projects.nitrc.org/indi/retro/atlas.html (2021).

[42] Liew, S.-L. *et al*. The Anatomical Tracings of Lesions after Stroke (ATLAS) Dataset - Release 2.0, 2021 (ICPSR 36684). *The Archive of Data on Disability to Enable Policy and research (ADDEP)*10.3886/ICPSR36684.v4 (2021).

[43] Kégl, B. *et al*. The RAMP framework: from reproducibility to transparency in the design and optimization of scientific workflows. (2018).

[44] Gau, R. *et al*. Brainhack: Developing a culture of open, inclusive, community-driven neuroscience. *Neuron* **109**, 1769–1775 (2021). 33932337 10.1016/j.neuron.2021.04.001 PMC9153215

[45] Winzeck, S. *et al*. ISLES 2016 and 2017-benchmarking ischemic stroke lesion outcome prediction based on multispectral MRI. *Frontiers in neurology* **9**, 679 (2018). 30271370 10.3389/fneur.2018.00679 PMC6146088

[46] Hakim, A. *et al*. Predicting Infarct Core From Computed Tomography Perfusion in Acute Ischemia With Machine Learning: Lessons From the ISLES Challenge. *Stroke*, STROKEAHA. 120.030696 (2021). 10.1161/STROKEAHA.120.030696 PMC8240494 33957774

